# Cdc42 regulates apical membrane fusion via the Rab11a–VAMP2 pathway in salivary gland acinar cells

**DOI:** 10.1101/2025.07.31.667845

**Published:** 2025-08-01

**Authors:** Akiko Shitara, Haruna Nagase, Christopher K. E. Bleck, Yuta Ohno, Hideo Kataoka, Keitaro Satoh, Masanori Kashimata

**Affiliations:** 1 Department of Biology, Asahi University School of Dentistry, 1851-1 Hozumi, Mizuho, Gifu 501-0296, Japan.; 2 Department of Pharmacology, Asahi University School of Dentistry, 1851-1 Hozumi, Mizuho, Gifu 501-0296, Japan.; 3 Electron Microscopy Core Facility, National Heart Lung and Blood Institute, National Institutes of Health, Bethesda, MD 20892, USA.; 4 Howard Hughes Medical Institute, Janelia Research Campus, Ashburn, VA 20147, USA; 5 Department of Basic Education, Asahi University School of Dentistry, 1851-1 Hozumi, Mizuho, Gifu 501-0296, Japan.

**Keywords:** Cdc42, Rab11a, VAMP2, apical membrane, apical recycling endosome, salivary gland

## Abstract

Epithelial polarity is essential for proper tissue organization and function, yet the molecular mechanisms governing apical membrane formation during secretory epithelial development remain incompletely understood. Here, we investigate the role of the small GTPase Cdc42 in salivary gland acinar cell development using a mouse model designed to knock out Cdc42 specifically at the onset of acinar cell formation. Loss of Cdc42 resulted in defective apical membrane formation accompanied by accumulation of vesicles around the apical lumen. These vesicles contained the apical water channel AQP5 and the apical recycling endosome (ARE) marker Rab11a, while the basolateral transporter NKCC1 retained normal localization, indicating an apical-selective trafficking defect. Importantly, Cdc42 deficiency caused a selective 40% reduction in the expression of the SNARE protein VAMP2, while other vesicle trafficking proteins including VAMP8, SNAP23, and EEA1 remained unchanged. Our findings reveal that Cdc42 controls apical membrane formation by maintaining VAMP2 expression, which is essential for the fusion of Rab11a-positive recycling endosomes. The accumulation of fusion-incompetent AREs near the apical surface demonstrates the critical role of the Cdc42-VAMP2 pathway in epithelial development. These results provide new insights into how polarity regulators integrate vesicle trafficking and fusion machinery, and may have implications for understanding glandular diseases involving epithelial polarity defects.

## Introduction

Epithelial cells establish tissue integrity through apical-basal polarity. This polarity enables directional transport of proteins and solutes, which is essential for organ function[1,2]. In secretory epithelia such as salivary glands, acinar cells must form polarized structures during development. These structures have distinct apical membrane domains and basolateral membrane domains [3,4]. Proper formation of these polarized structures requires precise coordination of membrane transport pathways. However, the control mechanisms remain poorly understood in vivo.

Small GTPases orchestrate membrane trafficking and epithelial polarity[5,6]. Among them, the Rho-family GTPase Cdc42 is a key upstream regulator: it activates the Par6–aPKC complex, remodels the actin cortex, and guides apical transport in intestine, kidney and other epithelia[7,8]. Emerging evidence further indicates that Cdc42 activity facilitates the delivery of Rab11a-positive apical recycling endosomes (AREs) [9]. Rab11a, in turn, serves as a marker of AREs. These organelles shuttle newly synthesised and recycled cargo to the nascent luminal membrane, thereby expanding the apical domain during morphogenesis; accordingly, Rab11a is indispensable for apical membrane biogenesis [10,11].

Vesicle fusion at this surface relies on SNARE complexes; in secretory epithelia both AREs and secretory granules are believed to converge on the lumen via VAMP2- or VAMP8-containing SNAREs [12–14]. Yet how loss of an upstream regulator like Cdc42 perturbs this Rab11a–SNARE regulatory pathway *in vivo* remains unknown.

Salivary gland acinar cells develop polarized apical and basolateral domains, together with secretory competence, during late embryonic and early postnatal stages [15]. We previously showed that acinar-specific deletion of Cdc42 disrupts apical membrane formation, lumen morphogenesis, and subsequent lumen maintenance [16,17]. However, the specific molecular pathways remain unclear.

In this study, we used a mouse model that specifically disrupts Cdc42 at the onset of acinar cell formation to investigate how Cdc42 regulates apical membrane formation. Here, we demonstrate that Cdc42 orchestrates apical membrane formation in developing acinar cells primarily by maintaining VAMP2 levels and enabling Rab11a-endosome fusion, thereby uncovering a Cdc42–Rab11a–SNARE regulatory pathway critical for secretory-gland morphogenesis.

## Materials and Methods

### 1. Mouse Models and Ethical Approval

All animal procedures were approved by the Animal Experiment Committee of Asahi University (approval numbers: 23–025, 24–029, 22–011) and conducted in accordance with institutional guidelines.

mT/mG reporter mice were obtained from The Jackson Laboratory (Bar Harbor, ME, USA). Cdc42^flox/flox^ mice and ACID-Cre (AQP5-Cre) mice were kindly provided by Dr. Y. Zheng (Cincinnati Children’s Hospital Medical Center, OH, USA) [18] and Dr. Z. Borok (University of Southern California, CA, USA) [19], respectively.

To generate acinar cell-specific Cdc42-deficient mice, Cdc42^flox/flox^; AQP5-Cre^+^; mT/mG mice were generated by crossing the respective lines as previously described [16,17]. Both male and female mice aged 10–35 weeks and weighing 20–40 g were used. Animals were housed under standard conditions (12 h light/dark cycle, 22 ± 2°C, 50 ± 10% humidity) with ad libitum access to food and water.

### 2. Tissue Collection and Processing

Mice were anesthetized by intraperitoneal injection of ketamine (100 mg/kg) and xylazine (20 mg/kg), followed by transcardial perfusion with 4% paraformaldehyde (PFA) in phosphate-buffered saline (PBS). Parotid glands were harvested and post-fixed overnight in 4% PFA at 4°C. Tissues were cryoprotected in 15% and 30% sucrose in PBS, embedded in OCT compound (Sakura Finetek, Torrance, CA, USA), and frozen in 2-methylbutane cooled with dry ice. Cryosections (10 μm) were prepared using a CM1850 cryostat (Leica Biosystems, Wetzlar, Germany) for immunostaining.

### 3. Immunofluorescence Staining and Confocal Imaging

Sections were permeabilized and blocked in PBS containing 0.02% saponin and 10% fetal bovine serum (FBS). Primary antibodies were applied overnight at 4°C.

The following day, Alexa Fluor-conjugated secondary antibodies were applied for 45 min at room temperature. Phalloidin-405 was used for F-actin visualization when applicable. Details of all antibodies are listed in Supplementary Table S1. Slides were mounted with Fluoromount (Diagnostic BioSystems, Pleasanton, CA, USA) and imaged using an LSM980 confocal microscope (Carl Zeiss, Oberkochen, Germany) with Zen software. For comparative analysis, control (Cdc42^+^) and Cdc42-deficient samples were imaged under identical acquisition parameters, and images were displayed with the same brightness and contrast settings. Images were analyzed using Imaris (Oxford Instruments, Abingdon, UK) and Fiji (ImageJ).

### 4. Transmission Electron Microscopy

Salivary glands were fixed for 90 min in 0.1 M phosphate buffer (pH 7.2) containing 2% glutaraldehyde and 2% PFA (Electron Microscopy Sciences, Hatfield, PA, USA). Post-fixation with aqueous 1% osmium tetroxide and *en bloc* staining with 1% uranyl acetate were performed. Samples were dehydrated in graded ethanol and embedded in EMbed-812 resin (Electron Microscopy Sciences).

Ultrathin sections were stained with uranyl acetate and lead citrate and examined with a JEM-1200EX transmission electron microscope (JEOL, Tokyo, Japan) at 80 kV. Micrographs were captured with a bottom-mounted 6-megapixel CCD camera (Advanced Microscopy Techniques, Woburn, MA, USA).

### 5. Western Blotting

Parotid glands were dissected from anesthetized mice, homogenized in RIPA buffer containing protease/phosphatase inhibitors and 0.5 mM PMSF, and incubated on ice for 15 min. Lysates were centrifuged at 14,000×g (4°C, 10 min), and supernatants were collected. Protein concentration was determined via the Bradford assay. Equal amounts (20 μg) were resolved by SDS-PAGE and transferred to PVDF membranes (Trans-Blot Turbo, Bio-Rad, Hercules, CA, USA).

Membranes were incubated in 10% skim milk prepared in TBS-T at 4°C overnight for blocking, followed by antibody incubation using an enhanced rapid immunoblotting protocol as described previously [20,21]. Primary antibodies were diluted in Can Get Signal^®^ Solution 1 (Toyobo, Osaka, Japan), and secondary antibodies conjugated with horseradish peroxidase (HRP) were diluted in Can Get Signal^®^ Solution 2. Protein bands were visualized using enhanced chemiluminescence (ECL) reagent (Cytiva, Marlborough, MA, USA).

Band intensities were quantified using Fiji and normalized to GAPDH. Experiments were performed on tissues from n = 7 Cdc42^+^ control and n = 7 Cdc42-deficient mice. See Supplementary Table S1 for antibody details.

### 6. Image Quantification: Distance Measurement from GFP Clusters to Lumen

Imaris software was used to generate 3D surface models for GFP-positive clusters and lumens. The distance between clusters and lumen surfaces was computed using the “shortest distance calculation” function. Thresholds were determined visually.

### 7. Statistical Analysis

All data are presented as mean ± SEM. Comparisons between two groups were performed using the two-tailed unpaired two-tailed Student’s t-test. Statistical significance was defined as *p* < 0.05.

All analyses were performed using GraphPad Prism version 7.0 (GraphPad Software, San Diego, CA, USA, www.graphpad.com)

## Results & Discussion

### Loss of Cdc42 leads to accumulation of vesicles near the apical lumen in salivary gland acinar cells

**Figure 1.**
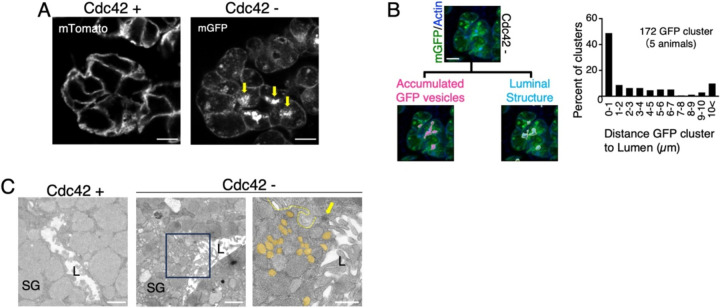
Cdc42 deletion causes vesicle crowding beneath the apical surface (A) Representative confocal images of parotid gland tissue from control (Cdc42^+^) and acinarspecific Cdc42 knockout (Cdc42^ΔAcini^, Cdc42^−^) mice. Plasma membranes are labelled by mTomato (red) or Cre-activated mGFP (green); yellow arrows highlight mGFP-positive vesicle clusters Scale bar, 10 μm. (B) Spatial analysis of vesicle-to-lumen distance. *Left*, workflow schematic: lumina are stained with phalloidin-405 (blue), mGFP clusters (green) and the luminal surface (cyan) are rendered in Imaris, and the shortest vesicle–lumen distances are calculated (magenta arrows). *Right*, histogram of distance distribution for 172 mGFP clusters collected from five Cdc42^−^mice. Scale bar, 10 μm. (C) Transmission-electron micrographs of acinar cells. *Left*, control gland (Cdc42^+^). *Middle*, Cdc42^−^ gland showing vesicle accumulation near the lumen. *Right*, enlargement of the boxed region in the middle panel; vesicles ≤200 nm in diameter are pseudo-coloured orange, cell boundaries are traced by yellow dashed lines, and a tight junction is marked by a yellow arrow. SG, secretory granule; L, lumen. Scale bars, 1 μm (left, middle) and 500 nm (right).

To explore the role of Cdc42 in membrane organization, we generated acinar cell-specific Cdc42 knockout mice by crossing ACID-Cre mice with Cdc42^flox/flox^ mice [16,17]. This mouse model was engineered so that Cdc42 knockout is initiated precisely at the onset of acinar cell formation, which begins during embryonic development. Cre recombinase activity was visualised using the mT/mG reporter system [22]. In Cdc42-deficient cells, membrane-targeted GFP localised not only to the plasma membrane but also to discrete intracellular vesicles. These GFP-positive vesicles did not exhibit a random cytoplasmic distribution; instead, they were enriched in regions adjacent to the apical surface (Figure 1A). Three-dimensional reconstruction confirmed that approximately 50% of the GFP cluster was located within 1 μm of the apical lumen (Figure 1B, C). Transmission-electron microscopy showed numerous small vesicles (approximately 0.1–0.25 μm) clustered near the luminal membrane (Figure 1C, middle and right panels; orange). In contrast, comparable vesicles were absent from Cdc42^+^ control glands (Fig. 1C, left).

The accumulation of vesicles near the apical lumen mirrors earlier 3-D culture findings in which Cdc42 loss disrupted apical membrane identity and vesicle targeting [9,23]. Importantly, our tissue-based observations reveal that these GFP-positive vesicle clusters are located within approximately 1 μm of the apical surface. This spatial constraint suggests that Cdc42 acts primarily in the final stages of vesicle–membrane fusion rather than in long-range trafficking events.

### Cdc42 loss causes mislocalization of apical AQP5/Rab11a while preserving basolateral NKCC1

**Figure 2.**
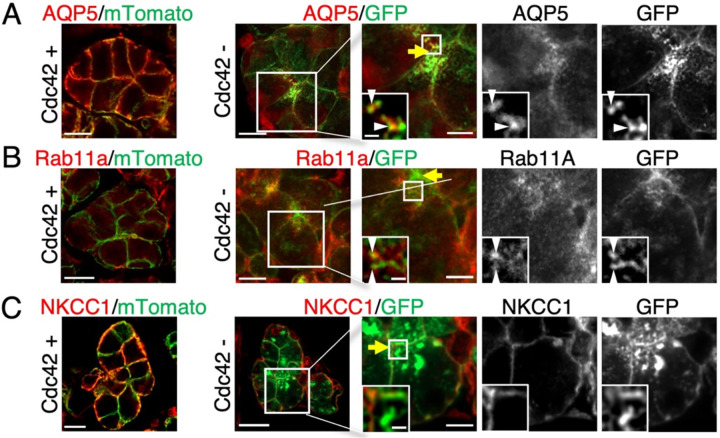
Mislocalization of AQP5 and Rab11a in Cdc42-deficient cells (A–C) Immunostaining for AQP5, Rab11a and NKCC1 (red) together with mGFP/mTomato (green). In Cdc42^−^ cells, AQP5 and Rab11a accumulate in vesicle-like clusters adjacent to the lumen (arrowheads), whereas NKCC1 retains basolateral localisation. Arrowheads indicate representative examples of vesicles showing colocalization of GFP with AQP5 or Rab11a. Yellow arrows mark the apical surface; insets show enlargements. Scale bars, 10 μm; insets, 1 μm. See Supplementary Table S1 for antibody details.

To determine the identity of vesicles accumulating near the apical lumen in Cdc42-deficient salivary gland acinar cells, we examined the localization of AQP5, a water channel predominantly localized to the apical plasma membrane [15,24], and Rab11a, a small GTPase that labels apical recycling endosomes (AREs) essential for apical membrane biogenesis [10]. In Cdc42^+^ control acinar cells, AQP5 showed its expected apical and basolateral distribution, whereas Rab11a was distributed throughout the cytoplasm (Figure 2A, B). In contrast, Cdc42-deficient cells showed a striking mislocalization of both proteins: AQP5 and Rab11a both accumulated in vesicle-like structures near the apical surface. These structures partially overlapped with mGFP-positive vesicles visualized by the mT/mG reporter, indicating that Cdc42 deficiency causes ectopic accumulation of membrane trafficking vesicles near the apical domain. Importantly, the basolateral transporter NKCC1 [25] retained its normal localization even in Cdc42-deficient cells (Figure 2C). This selective effect demonstrates that Cdc42 specifically regulates apical transport pathways.

The co-accumulation of AQP5 and Rab11a in the same vesicles suggests that these represent AREs that have failed to fuse with the luminal membrane. This interpretation is consistent with the established role of Rab11a in mediating recycling from endosomes to the apical surface [10]. Indeed, Rab11a knockout mice have been reported to show accumulation of apical proteins in subapical regions [11], similar to the vesicle accumulation near the lumen that we observed. These findings collectively demonstrate that Cdc42 is essential for proper apical membrane trafficking, specifically affecting the distribution of AREs.

### Cdc42 deficiency selectively reduces VAMP2 expression, suggesting impaired vesicle fusion

**Figure 3.**
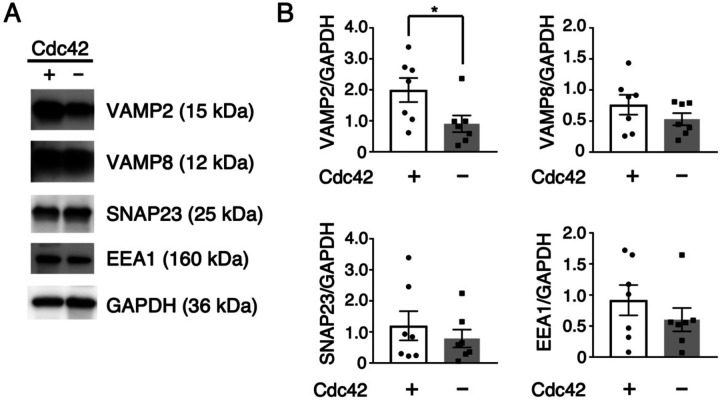
VAMP2 expression is downregulated in Cdc42-deficient glands (A) Immunoblots of VAMP2, VAMP8, SNAP23 and EEA1 in parotid lysates from control and Cdc42^−^ mice; GAPDH is the loading control. (B) Densitometry (mean ± SEM, *n* = 7). VAMP2 is reduced by ~40 % in Cdc42^−^ glands (*p* < 0.05, two-tailed unpaired *t*-test), whereas VAMP8, SNAP23 and EEA1 are unchanged. Antibody information is provided in Supplementary Table S1.

The accumulation of AQP5/Rab11a-positive vesicles could result from either impaired fusion with the apical membrane or excessive endocytosis from the apical surface. To distinguish between these two potential mechanisms, we examined the expression levels of key proteins involved in vesicle fusion and endocytosis. We analyzed SNARE proteins that regulate fusion events (VAMP2, VAMP8, SNAP23)[26] and EEA1, which controls endocytic processes[27]. Western blot analysis revealed that VAMP2 expression was significantly decreased in Cdc42-deficient parotid glands, with an approximate 40% reduction relative to GAPDH (Figure 3A, B). In contrast, the expression levels of VAMP8, SNAP23, and EEA1 remained unchanged between Cdc42^+^ control and Cdc42-deficient cells.

The selective reduction in VAMP2, with no changes in endocytic machinery (EEA1) or other SNARE proteins, suggests that Cdc42 deficiency primarily impairs vesicle fusion rather than promoting excessive endocytosis. Furthermore, this specificity is particularly notable because both VAMP2 and VAMP8 function in apical membrane fusion during exocytosis, yet only VAMP2 is affected by Cdc42 loss. The reduction in VAMP2 levels provides a molecular explanation for the vesicle accumulation phenotype. With diminished VAMP2, AREs are transported normally to the apical region but fail to fuse with the luminal membrane, resulting in their accumulation near the apical surface.

### Role of the Cdc42-VAMP2-Rab11a pathway in acinar cell development and homeostasis

Our findings collectively support a model in which Cdc42 controls apical membrane formation during acinar cell development primarily through maintaining VAMP2 expression and promoting fusion of Rab11a-positive recycling endosomes. We propose that Cdc42 ensures fusion competence of apical recycling endosomes by protecting VAMP2 expression. This regulatory mechanism is important for proper construction of the apical membrane and lumen during acinar cell development. The Cdc42 knockout in our study begins from the early stages of acinar cell formation, and the observed phenotypes reflect the role of Cdc42 in acinar structure formation during development. We have previously shown that knockout of Cdc42 from mature acinar cells results in luminal morphological changes[17], demonstrating that Cdc42 is important for maintaining luminal membrane homeostasis. This VAMP2-Rab11a pathway may contribute to both acinar formation during development and maintenance of luminal membrane homeostasis in mature acinar cells.

A key limitation of our study is that how Cdc42 regulates VAMP2 remains unclear. While our data demonstrate that Cdc42 deficiency reduces VAMP2 protein levels, the underlying mechanism—whether through transcriptional control, protein stability, or SNARE complex regulation—is unknown. Additionally, it will be important to determine whether this Cdc42-VAMP2 regulatory relationship is conserved across different secretory epithelia and whether its disruption contributes to disease pathogenesis. Such studies would help establish the broader relevance of this mechanism in epithelial biology and human disease.

In conclusion, this study identifies Cdc42 as a central coordinator of apical membrane formation during acinar cell development through maintenance of VAMP2 expression and regulation of ARE fusion. These findings provide new insights into how polarity regulators control membrane trafficking and may have implications for understanding epithelial disorders in secretory tissues.

## Supplementary Material

Supplement 1

## Figures and Tables

**Figure 4.**
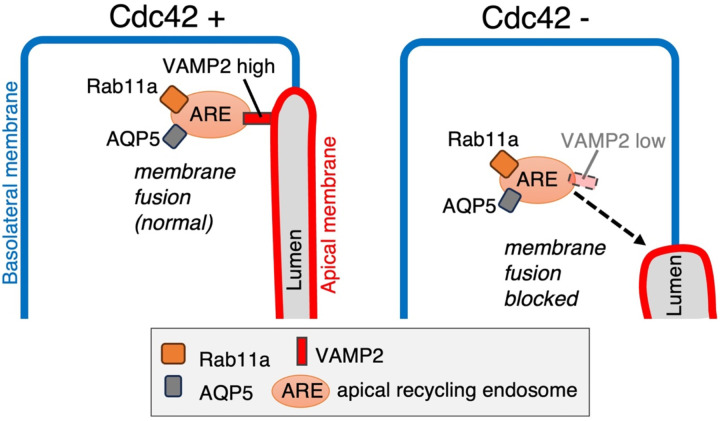
Proposed Cdc42-centred model for apical trafficking in salivary acinar cells **Left, Cdc42**^**+**^: Rab11a-positive apical recycling endosomes (ARE) that carry the water channel AQP5 dock beneath the apical membrane; Cdc42 maintains high VAMP2 levels on these vesicles, allowing SNARE-mediated fusion with the apical membrane. **Right, Cdc42**^−^: Loss of Cdc42 lowers VAMP2, causing ARE-derived vesicles to stall and fail to fuse; AQP5 and Rab11a accumulate in the sub-apical region and luminal delivery is blocked. Blue lines, basolateral membrane; red lines, apical membrane. Symbol key (legend box): orange, Rab11a; grey, AQP5; red, VAMP2; salmon, ARE.
